# An Evaluation of the Process and Quality Improvement Measures of the University of Virginia Cancer Center Tobacco Treatment Program

**DOI:** 10.3390/ijerph17134707

**Published:** 2020-06-30

**Authors:** Kara P. Wiseman, Lindsay Hauser, Connie Clark, Onyiyoza Odumosu, Neely Dahl, Jennifer Peregoy, Christina W. Sheffield, Robert C. Klesges, Roger T. Anderson

**Affiliations:** 1 Department of Public Health Sciences, School of Medicine, University of Virginia, Charlottesville, VA 22908, USA; rck2n@virginia.edu (R.C.K.); ra2ee@hscmail.mcc.virginia.edu (R.T.A.); 2 Cancer Center, University of Virginia, Charlottesville, VA 22908, USA; lh7yn@hscmail.mcc.virginia.edu (L.H.); cmc4nv@hscmail.mcc.virginia.edu (C.C.); oo2c@virginia.edu (O.O.); nd4h@hscmail.mcc.virginia.edu (N.D.); jap7qv@hscmail.mcc.virginia.edu (J.P.); cws9q@hscmail.mcc.virginia.edu (C.W.S.); 3 Center for Addiction Prevention Research, University of Virginia, Charlottesville, VA 22908, USA

**Keywords:** Cancer survivorship, smoking cessation, quality improvement, process mapping

## Abstract

Tobacco use after a cancer diagnosis can increase risk of disease recurrence, increase the likelihood of a second primary cancer, and negatively impact treatment efficacy. The implementation of system-wide comprehensive tobacco cessation in the oncology setting has historically been low, with over half of cancer clinicians reporting that they do not treat or provide a referral to cessation resources. This quality improvement study evaluated the procedures for assessing and documenting tobacco use among cancer survivors and referring current smokers to cessation resources at the University of Virginia Cancer Center. Process mapping revealed 20 gaps across two major domains: electronic health record (EHR), and personnel barriers. The top identified priority was inconsistent documentation of tobacco use status as it impacted several downstream gaps. Eleven of the 20 gaps were deemed a high priority, and all were addressed during the implementation of the resulting Tobacco Treatment Program. Prioritized gaps were addressed using a combination of provider training, modifications to clinical workflow, and EHR modifications. Since implementation of solutions, the number of unique survivors receiving cessation treatment has increased from 284 survivors receiving cessation support during Year 1 of the initiative to 487 in Year 3. The resulting Tobacco Treatment Program provides a systematic, personalized, and sustainable comprehensive cessation program that optimizes the multifaceted workflow of the Cancer Center and has the potential to reduce tobacco use in a population most in need of cessation support.

## 1. Introduction

Tobacco use is associated with at least 15 types of cancer and accounts for 30% of all cancer deaths. Yet, 15% of adults in Virginia are current smokers, with regional variation ranging from 10–24% [1]. The University of Virginia (UVA) Cancer Center, a National Cancer Institute (NCI)-designated Cancer Center for over 30 years, provides care to a diverse patient population from 87 counties in Virginia and eastern West Virginia. Many of the counties are non-metropolitan and rural, which report some of the highest rates of tobacco use in Virginia. Smokers in rural areas also smoke more cigarettes per day, are more likely to be dual and poly tobacco users, and initiate tobacco use at an earlier age than those in non-rural areas [2,3,4,5]. As a result, cancer patients (hereafter referred to as cancer survivors) living in these regions may be highly addicted to nicotine and yet have limited access to cessation services in their local communities [5]. In response, cancer centers are often looked at to provide cessation services to support survivors. In 2018, UVA was involved in the care of roughly 4,000 cancer survivors; in 2019, among all patient encounters, about 11% of survivors self-reported current tobacco use.

Providing consistent and effective cessation in the cancer setting is critical [6]. Continued tobacco use after a cancer diagnosis can increase disease recurrence, increase the likelihood of a second primary cancer, and negatively impact treatment efficacy [7]. Importantly, quitting tobacco use can have significant benefits including improved quality of life and increased survival [8]. A cancer diagnosis can increase a person’s motivation and interest in tobacco cessation and creates a potential opportunity for oncology clinicians to engage and offer cessation services [9]. To that end, the American Society for Clinical Oncology recommends assessing tobacco use status at every clinic visit given the likelihood in changes motivation and relapse [10]. Even with the known benefits of cessation and national recommendations supporting cessation in oncology care, consistent cessation is not always provided [11]. Specifically, less than half of oncology clinicians actively treat or refer their patients to cessation services [12]. Barriers at multiple levels, including providers’ lack of knowledge of existing intervention resources, a lack of easy referral systems, and trouble identifying smokers, may explain some of the low utilization of cessation services in oncology settings [11]. More thorough integration of tobacco control activities in oncology settings provides an opportunity to better identify current tobacco users and provide consistent, tailored treatment based on personal factors and cancer treatment plan, which would result in higher cessation rates and reduced negative implications from continued tobacco use during and after oncology care [13].

As a part of the NCI Cancer “Moonshot Program”, the UVA Cancer Center was one of 42 cancer centers funded as part of the Cancer Center Cessation Initiative (C3I) to build and implement a sustainable tobacco cessation treatment program for cancer survivors at any treatment stage [6]. Herein, we describe a quality improvement study conducted using Lean methodology, implemented as part of the C3I at the UVA Cancer Center and describe the resulting Tobacco Treatment Program (TTP).

## 2. Materials and Methods

### 2.1. Setting

The current quality improvement study assessed existing procedures for determining and documenting tobacco use, referring tobacco users to cessation resources, and implementation of cessation services at the Emily Couric Clinical Cancer Center (ECCCC) through a six-month comprehensive process. UVA’s participation in the C3I and subsequent initiation of the quality improvement began in October 2017. The UVA Health System does not require Institutional Review Board (IRB) approval for assessments of quality improvement processes and implementation of new clinical practice. Therefore, IRB approval was not required as part of the UVA C3I.

### 2.2. Approach

Lean methodology aims to improve a process by maximizing value for patients through the elimination of non-value adding activities while involving all employees in the process of identifying and eliminating those non-value adding activities [14,15]. The five principles of Lean methodology are: identify value, map the process, create flow, establish pull, and seek perfection. It is an ongoing process with no specific end point, and no requirement to “complete” the five principles. The first principle, “identify value” involves identifying the customer and the value of the service. In health-care settings, value is defined from the perspective of the patient [16]. Within the UVA C3I, the “customers” were current tobacco users and the service was the TTP or cessation. The value is the health of the survivor through cessation. The second principle, “map the process” defines the existing process, which assists in identifying gaps that need to be addressed. The third principle, “create flow” focuses on developing solutions to maximize efficiency and service, reduce waste, and achieve program goals. The fourth principle, “establish pull”, is focused on creating demand and sustained interest in the program. The fifth principle, “seek perfection” stresses the importance of iteration and growth of program implementation, capturing the need to continue to develop and grow. The UVA C3I encompasses all five principles. However, the quality improvement study was primarily guided by “identify value”, “map the process”, and “create flow”, which are describe in more detail below.

#### 2.2.1. Map the Process

**Figure 1:**
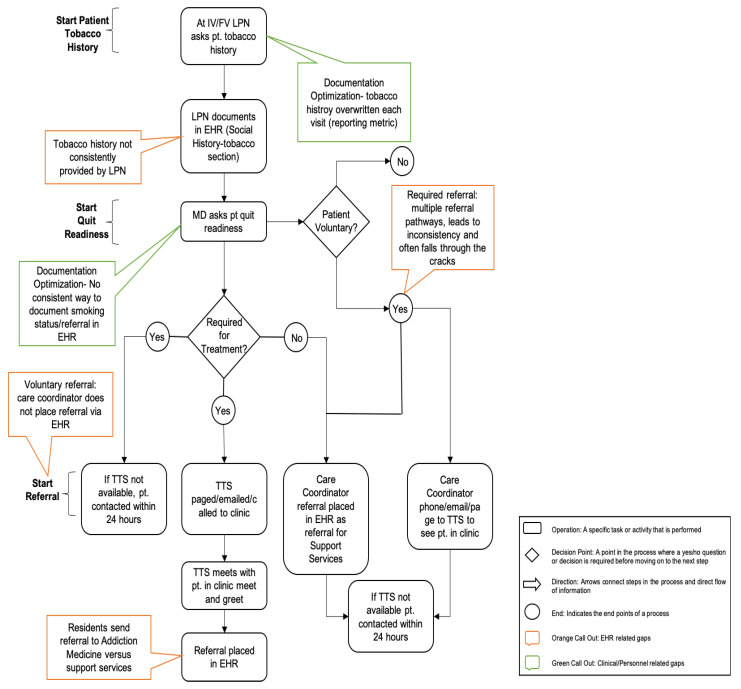
Snapshot of Value Stream Map. IV = initial visit, FV = follow-up visit, LPN = licensed practical nurse, pt = patient, MD = physician, TTS = Tobacco treatment Specialist, EHR = Electronic Health Record.

Lead by a Quality Improvement Manager and guided by the C3I team, the process phase separated the TTP into phases starting with a cancer survivor arriving to the cancer clinic through their follow up visits for the TTP. Specifically, the Tobacco Treatment Specialist (TTS) for the TTP as the subject matter expert and the Manager for Cancer Support Services (leadership), provided the Quality Improvement Manager a detailed description of the TTP, including all steps, stakeholders, decisions, and potential endpoints. The Quality Improvement Manager took this detailed description of the process and created a value stream map using LUCID chart software (Figure 1). The goal of the value stream map was to separate the process into discrete segments. The value stream map used unique identifiers for actions that occurred during the process, movement between processes, question/decision points, end points, and identification of potential or known gaps in the process. The process review also included the Quality Improvement Manager performing a Gemba walk, which is observing the actual process, engaging with employees, gaining knowledge about the work process, and exploring opportunities to improve [17]. During the Gemba walk the Quality Improvement Manager observed the process and validated the value stream map by shadowing and asking questions with the TTS, rooming staff and patient schedulers. Throughout this stage, the value stream map was reviewed with the C3I team for correction and fine-tuning. The process review concluded by categorizing the identified gaps (examples of where gaps are located in the value-stream are depicted below in call-out boxes). Within the UVA C3I, two categories were identified: electronic health record (EHR) system and clinical personnel.

#### 2.2.2. Create Flow

Following process mapping and identification of gaps, each one was reviewed and prioritized, and potential solutions began to be developed. Gap prioritization focused on those that most reduced the value of the TTP, were significantly limiting efficiency, or had important downstream effects to other identified gaps. For some gaps, solutions were readily available. For gaps where additional information was needed, an “A3” was conducted. An A3 is a concise method used to define a problem, with the name being derived from the goal of being able to summarize the problem on a A3 piece of paper (11.7 × 16.5 inches) [18]. The A3 defines one problem at a time in the most structured way possible to facilitate the development of solutions. It can include verbal or visual aids and provide easily digested information. An A3 was created for many gaps, however, it was primarily used for solution development for the highest priority gap, tobacco use documentation in the EHR. Specifically, departmental reports were run to examine tobacco encounters at the survivor level for each clinic visit at the ECCCC. The total number of encounters (e.g., visits) was compared to the total number of updated tobacco histories. Additionally, the Quality Improvement Manager performed additional Gemba walks by shadowing of the TTS and Licensed Practical Nurses (LPNs, n = 3), as well as rounding in clinics, engaging, and surveying front line staff. These observations revealed additional gaps and allowed for discussion of potential solutions between the C3I team and others who would be directly impacted by process changes. The A3 concluded by providing a structured, operationalized definition of the problem, potential solutions, and definitions to document impact of potential solutions. Potential solutions were discussed by the C3I team. During this process, the TTS presented and discussed potential solutions with multiple stakeholder groups (patient schedulers, care coordinators, tumor board and core staff meetings). The TTS also participated in training (if applicable) during implementation of final solutions.

### 2.3. Data and Analyses

Data to quantitatively assess TTP implementation and outcomes was provided using EHR reports. Reports were pulled quarterly starting in 2017 and included the number of adult survivor visits at the ECCCC, the number of survivors with documented tobacco use status, the number of current cigarette smokers, and the number of survivors who enrolled in the TTP. Among survivors who enroll in the TTP, a second EHR report provides information on self-reported quit rates and number of cigarettes smoked per day using the most recently reported data.

Assessment rates were defined as the number of survivors who had tobacco use status documented/number of survivors seen in the ECCC over the same reporting period. Quitting smoking was defined as the number of survivors who reported quitting smoking completely/number of survivors enrolled in the TTP over the same reporting period. Reduction in smoking is associated with subsequent cessation; thus, this metric is an important prognostic indicator of future smoking cessation [19] and was defined as the number of survivors who reported reducing their number of cigarettes smoked per day by at least 50%/number of survivors enrolled in the TTP over the same reporting period.

## 3. Results

### 3.1. Gap Analysis and Prioritization

The gap analysis identified 20 gaps across the two major domains (EHR/personnel, Table 1). Personnel gaps were further divided between clinician and Center-level. Specifically, of the 20 identified gaps, 45% (n = 9) were EHR gaps, 45% (n = 9) were Center gaps, and 10% (n = 2) were clinician or clinician/Center gaps. An EHR-related gap was the absence of an automatic referral process for tobacco treatment within the EHR, making it more time consuming to manually create each referral. Under the domain of clinician gaps, LPN staff responsible for assessing tobacco use had not received an orientation or training on how to assess this behavior. A Center-level gap included a lack of orientation for nurses and residents/fellows about the TTP and existing workflow. Among the 20 identified gaps, 11 were identified as high priority. Among those identified as a high priority, 36% (n = 4) were EHR gaps, 46% (n = 5) were Center/workflow level gaps, and 18% (n = 2) were clinician or clinician/Center gaps.

### 3.2. Development of Solutions and Implementation of New Program

#### 3.2.1. Tobacco Use Documentation

As a result of the gap analysis, the top identified priority was inconsistent documentation of tobacco use status in the social history section of the EHR. This lack of consistent documentation was a top priority because it had significant downstream effects on the program. For example, addressing priority 1 could also indirectly begin to address and correct priorities 2, 4, 6, and 7. It was hypothesized that the inefficiency in documentation could be either a system routing and/or flow error or a lack of training for staff around properly documenting social history, leading to several potential solutions. The A3 identified specific barriers including inconsistent documentation of cancer survivor tobacco use history between departments in the EHR, a lack of education around tobacco use and terms, and discomfort addressing tobacco use at every visit. With direction from the front-line staff, an updated standard of work and staff education tools were developed. This included a one-page quick reference guide highlighting what needed to be completed in the tobacco use history section of the EHR and definitions of the terms included in the tobacco use history section. In addition to the quick reference, the TTS joined monthly staff meetings to review the new standard work and engage in conversation around how to assess tobacco use at every clinic visit.

#### 3.2.2. Triaging

Through the gap analysis the efficiency of the current tiered triaging system was identified as not being optimally utilized by the referring providers. Providers would incorrectly refer survivors to a treatment tier because guidelines were not clear, and it was hard to differentiate between each tier. To simplify the tiered system, the model was adjusted to two tiers for survivors ready to quit and one tier for survivors not yet ready to quit (Table 2). In addition, tier 1, the most robust tier had clear clinical criteria that had to be met for a survivor to receive that level of care. Additionally, the number of sessions were modified to increase efficiency of the program based on the needs and interests of the survivor. The TTS spent two to three hours each week training physicians and care coordinators on the new system and criteria. Specifically, the TTS educated providers by attending tumor board, grand rounds, and staff meeting for all level of care providers. This training was important initially and as a continued process due to the nature of the UVA Cancer Center as an academic hospital with a continual rotation of medical students, residents and fellows as well as normal staff turnover. Prior to the implementation of the triaging improvements, 43% of survivors were referred as “urgent”. By the end of the year one of the C3I, urgent referrals were reduced to 19%.

#### 3.2.3. Flowsheets and Smart Phrases

The original TTP operated using paper tobacco use questionnaires and manual entry for survivor tracking. To improve tracking of survivor progression longitudinally and to standardize documentation the entire process, a “flowsheet” was developed within the EHR. The original paper questionnaire, which includes the Fagerström Test for Nicotine Dependence [20] and the Cancer Patient Tobacco Use Questionnaire [21], was asked periodically through a survivor’s time in the TTP, and was used as the foundation of the flowsheet with multiple tabs (e.g., independent entries) available per survivor. All treatment tiers had a tab for the initial visit in the TTP, all subsequent follow up visits/calls, and the 3-, 6- and 12-month follow up visits/calls. Within the flowsheet, “smart phrases” were developed with drop down choices and typical clinical reporting sentence structure for more consistent documentation of survivor encounters. For example, instead of documenting number of cigarettes smoked per day as part of a general comment, the smart phrase, “Current number of cigarettes per day?” with specific response options of “10 or fewer”, “11–20”, “21–30”, and “31 or more” was added as a specific question. Another smart phrase is “Potential barriers to quitting smoking” with response options, “Bad cravings”, “Boredom”, “Depression”, “lack of willpower”, “sudden impulses”, and “weight gain”. The flowsheet and smart phrases created a systematic progression of survivors through the program and allowed for streamlined reporting and improved communication of treatment plans with clinical partners; thus, addressed several other downstream priorities from the gap analysis.

### 3.3. Implementation of New Tobacco Treatment Program

The new program is as follows: tobacco use is assessed at each visit to the ECCCC by an LPN rooming nurse who is responsible for beginning the conversation about the TTP to all identified tobacco users. Information about survivors who are interested in cessation is sent to a care coordinator, who refers survivors to the TTP using an e-referral through the EHR. Survivors are triaged to designate immediacy of need for cessation services (Table 2). For survivors most in need of immediate cessation, a TTS is paged to attempt a same day appointment. Survivors who are not required to quit due to a medical procedure work with the Cancer Center scheduling staff to schedule an in-person initial visit with a TTS. After initiating the TTP, survivors are further classified based on readiness to quit smoking, which impacts the type of tobacco treatment they receive. In addition to referrals from the LPN rooming nurses who are responsible initiating a conversation about the TTP, physicians may refer survivors to the TTP, and survivors may self-refer at any time. After the TTP survivors in Tiers 1 and 2 receive cessation telephone counseling, with the number of calls varying depending on tier. Access to nicotine replacement therapy was included as part of the C3I.

**Figure 2:**
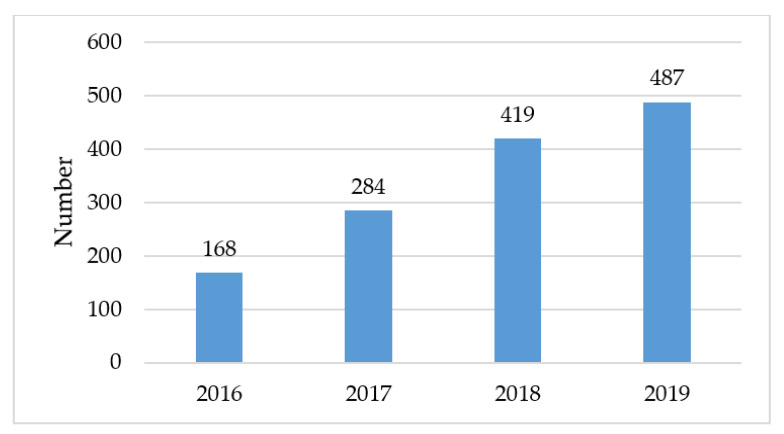
Unique Survivors Treated in the Tobacco Treatment Program at UVA during the C3I.

**Figure 3:**
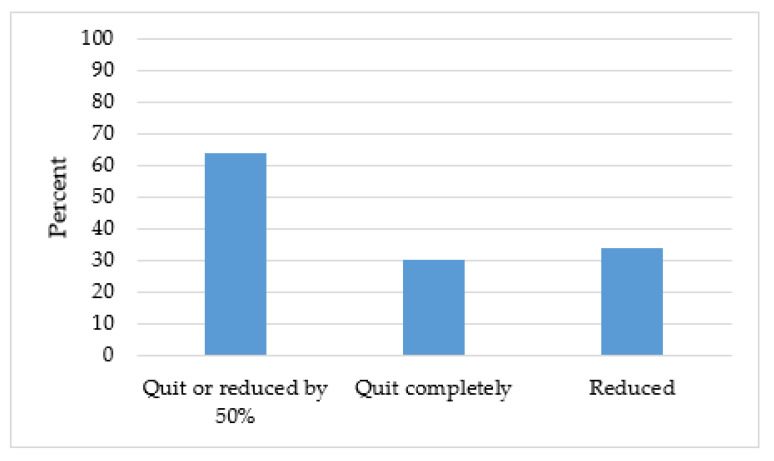
Self-reported quit and reduction rates among n = 211 survivors enrolled in the Tobacco Treatment Program. Reduced = reduced daily cigarettes by at least 50%.

Since the implementation of the new TTP, assessment of tobacco use is consistently implemented (99%). During the first year of the C3I initiative, 284 survivors received cessation support. The number of survivors receiving cessation treatment rose to 419 and 487 in Years 2 and 3, respectively (Figure 2). Among survivors for whom EHR flowsheet data are available (n = 211 survivors), 30% self-report having quit completely and 34% have reduced their number of cigarettes smoked per day by at least 50% (Figure 3).

## 4. Discussion

This quality improvement study used Lean methodology with A3 for gap identification and deep exploration of the TTP in the UVA Cancer Center. Multi-level gaps were identified and while a majority of identified gaps fell within the domains of the EHR and the Center, the prioritized solutions were more balanced between clinicians, Center, and EHR gaps. This highlights the interdependence between these elements of providing care, as one solution had the potential to impact gaps across domains, and how the gap analysis can assist in solution prioritization. Solutions also demonstrated the use of Lean methodology as several solutions were specifically designed to increase efficiency of the TTS. Specifically, implementing the updated tiered triaged system and staff training to improve correct tier designation, the number of high-risk survivors dropped significantly. With fewer survivor being referred as urgent, the TTS was paged less often for same day in-person clinic visits which optimized the TTS availability to treat more survivors. Additionally, the implementation of the TTP flowsheet and smart phrases reduced charting time for the TTS, which also increased availability to treat more survivors. By improving efficiency, the total number of patients served could increase without requiring additional personnel.

Lean methodology also provides maximum versatility for quality improvement. For example, other C3I sites have also used Lean methodology, using rapid experiments of process changes [22]. Rapid experimentation was not a component of the UVA C3I. The differences in use of specific lean methodology strategies might represent differences in how well-established each cancer center’s TTP was at the start of the C3I. For example, clinic settings that have well-established programs may be able to move quickly through process mapping and into experimentation, whereas newer programs might be better served by dedicating significant time to process mapping to identify the full suite of existing gaps. Importantly, assessment of tobacco use and use of tobacco treatment services increased dramatically in both cancer centers sites, showing the success that a personalized implementation of Lean methodology can have. Other clinical settings have employed lean methodology to increase delivery of cessation services and prescribing nicotine replacement therapy to cancer survivors using national survey data to inform Plan Do Study Act cycles [23]. 

The UVA C3I occurred within the context of 42 other funded C3I Cancer Centers located across the country. It is important to note that many of the C3I sites were located in and around urban centers across the country, while UVA Cancer Center is located in a small town with a predominately rural catchment area. This unique context in geography and culture is relevant when thinking about how to continue to build upon the success of the new TTP. For example, there are increased rates of dual and poly use among adults in rural areas [4], however at present, the TTP focuses primarily on smoking cessation (although a few poly-tobacco users have participated in the program). Thus, there is potential benefit of the UVA TTP more directly addressing multiple tobacco product use. In addition, survivors living in rural areas lack access to care and must sometimes travel great distances for care. This may make scheduling non-treatment related visits, such as the initial visit of the TTP, difficult for some survivors. Telemedicine is a potential solution, and one that has been used extensively during the COVID-19 pandemic. While medical centers are well-equipped to provide services via telemedicine, it must not be assumed that survivors have access to the equipment and internet bandwidth needed to receive care in this manner. The digital divide still exists in rural parts of the US [24], which is being considered as the TTP works towards expansion of services and balancing travel time with technology resources. For example, one possibility for telehealth to be effective in a rural area could be to partner with community locations that have telehealth facilities so that survivors have local access to high-speed internet connections and private facilities in which to receive care. Additionally, other types of tobacco counseling or intervention delivery that rely less on real-time video or internet voice communication may prove particularly relevant in this population (e.g., telephone or internet interventions). Lastly, the TTP developed at UVA uses a centralized model to provide care. This model could be useful for future implementation of cessation services beyond the Cancer Center. For example, the TTS within the Cancer Center could provide cessation services to current smokers referred from primary care or pulmonology clinics. A centralized model like the UVA TTP may also be an efficient way for community healthcare systems to provide consistent cessation services while sharing the cost of implementation.

The UVA C3I continues to employ Lean methodology focusing on “creating pull” and “seeking perfection”. For example, during the C3I project, funding for nicotine replacement therapies was included for those who did not have insurance. Therefore, as most survivors were able to receive medications, access to cessation medications was not an identified priority during the quality improvement study. However, after the implementation of the new TTP, the C3I identified the need to create a more efficient and sustainable pathway for those needing medication assistance. Prior to this phase, the TTS was responsible for all components of providing medication assistance to survivors and it was clear that this effort needed to be reduced to efficiency and sustainability. To realize this goal, a pre-scripted medication order was added to the EHR which sent an automated request for physician review and signature after the completion of the initial visit with the TTS. Additionally, the TTP partnered with the UVA Cancer Center pharmacy to include tobacco cessation medications as part their Pharmacy Patient Medication Assistance (PPMA) Program. As part of this program, a PPMA Flowsheet is completed by the TTS which then allows prescriptions to be sent to the main hospitals pharmacy to be filled. The PPMA team works through any prior authorizations that might be needed, connects survivors with prescription assistance programs, and provides other means possible to assist survivors with obtaining tobacco cessation products free or at a reduced cost.

The impact of quality improvement studies has been dramatically successful at increasing delivery of tobacco treatment to cancer survivors [22,23,25,26]. However, many of these studies have only examined short-term outcomes. It is less certain if these improvements will continue. Therefore, the use of a continuous quality improvement framework and sustainability is essential to realize the potential population health impact of comprehensive tobacco control within cancer centers [6]. While originally supported by C3I funded, the TTP has become systematically embedded into the Cancer Center making tobacco treatment an important part of all encounters with cancer survivors. By removing inefficiencies throughout the program, it is now is more sustainable. Resources, including staff time is maximized resulting in an increase of survivor referrals and appropriate triage to the program treatment levels. The time and effort used to build new EHR processes and flowsheets has created a system that is self-sufficient and provides the ability to scale-up if needed based on survivor load. The sustainable use of pharmacotherapy results in most survivors being able to receive medication therapy without requiring significant financial burden or TTP resources.

With a systematic and stable workflow now in place, the TTP has also begun to focus more on “creating pull”. The sustained implementation and regular evaluation of the new TTP provides an opportunity to learn more about tobacco cessation interventions that might prove particularly useful for cancer survivors and increase enrollment in the TTP. For example, even using an opt-out model recently implemented at the Mayo Clinic Cancer Center, only 17% of survivors attended a scheduled tobacco treatment appointment [25]. At the UVA Cancer Center, while assessment of tobacco use and referral to the TTP have both increased substantially, this increase has not produced an equivalent increase in survivor enrollment in the TTP. Thus, there continues to be an opportunity to increase the reach of tobacco cessation within cancer centers. Additionally, there is a need to maintain engagement with cessation, particularly as many smokers must make multiple attempts before successfully quitting. Future iterations of our TTP will attempt to modify existing services to better support smokers who relapse and evaluate its impact on long-term cessation. One potential barrier to increasing enrollment in TTP is the lack of repeat assessments of survivors’ readiness to quit. Singer et al. found that only 43% of tobacco using survivors assessed by a radiation oncologist about cessation expressed readiness to quit [26]. The myriad complexities (personal and medical) surrounding cancer treatment may mean that success in motivating a survivor to quit varies over time, reflecting a personal process of coping with a diagnosis of cancer, making it imperative that readiness to quit is frequently assessed and counseled. Providing additional resources or treatment options for smokers who are not ready to quit might prove particularly beneficial in a survivor population with heightened guilt and anxiety around smoking.

This study has several strengths and limitations that need to be considered. First, this quality improvement study focused on clinician, and EHR gaps to the implementation of comprehensive tobacco cessation within the UVA Cancer Center; as a result, cancer survivor barriers to cessation were not examined. However, continued evaluations of the current TTP could include patient reported outcomes and assessment of barriers to cessation. Second, this quality improvement study took place in one cancer center and as a result, findings may not be generalizable to other cancer centers or clinical settings. However, the implementation of Lean Methodology can occur regardless of the clinical setting and is designed to produce unique results by site. Third, as UVA Cancer Center is located in a small town, the total patient population may be lower than other academic medical centers located in more populous areas. However, the patient population served by UVA as a C3I site is relatively unique in that the catchment area includes many rural counties, which usually have a higher prevalence of tobacco use than non-rural areas. Lastly, the current study focused on the quality improvement process; thus, the study is more descriptive with a particular emphasis on lessons learned and identifying gaps. However, the new TTP is poised for future studies to undertake a formal program evaluation.

## 5. Conclusions

This article demonstrates the implementation of Lean methodology to evaluate and improve smoking cessation within UVA Cancer Center, reviewed top barriers, and described solutions that have resulted in a robust, comprehensive, and sustainable tobacco treatment program. By identifying the interdependence of identified gaps, it became clear that relatively small solutions had the potential for large downstream effects. The new TTP is poised to provide excellent care and serve as a platform for future cessation research with the potential to positively impact the health of survivors who use tobacco, particularly those living in rural areas.

## Figures and Tables

**Table 1:** Gap Analysis, Prioritization, and Potential Solutions.

Gap Number	Gap Description	Domain	Priority	Solution
1	Lack of LPN clarity on how to enter tobacco use information	EHR	High	Education/Training
2	“Mark as Reviewed” EHR button applied to all sections of Alcohol, Tobacco, and Other Drug Use Page, not just the Tobacco Use section and tobacco use updates overwrote previous entries (impacted by #1)	EHR	High	EHR
3	Inconsistent orientation to tobacco use assessment among LPNs	Center	High	Education/Training
4	No validation of process flow for current smokers (impacted by #1)	Center	High	Education/Training
5	No way to trigger automatic referral to TTS through the EHR	EHR	High	EHR
6	Fractured Ask, Advise, Connect (Impacted by #1)	Clinician/Center	High	Education/Training
7	Inconsistent use of TTP referral process using triage protocol (impacted by #1)	Center	High	Education/Training
8	Inconsistent orientation for nurse coordinators and residents/fellows for TTP referral standard work	Center	High	Education/Training
9	Accessibility of TTS (impacted by #7)	Clinician	High	Education/Training Workflow
10	Survivor information and progress within the TTP are manually entered in a non-standardized way	EHR	High	EHR
11	Non-optimized triaging (impacted by #7)	Center	High	Workflow Education/Training
12	Access to tobacco use history dependent on access level of staff	Center	Med	EHR
13	“Ready to Quit” EHR button “Yes” response not saved	EHR	Med	EHR
14	Referral data not available for validation or reporting to stakeholders (Clinical care team/ Administration/Grantors)	EHR	Med	EHR
15	Multiple referral queues (impacted by #8)	Center	Med	Education/Training, EHR
16	Lack of survivor communication tools (Telehealth)	Center	Med	Workflow
17	Outdated survivor education materials and standard work documentation	Center	Low	Workflow
18	No automated reminders to survivors for follow-up within TTP	EHR	Low	EHR
19	No automated referrals to State quit line or SmokefreeTXT	EHR	Low	EHR
20	Reports created manually	EHR	Low	EHR

Note: LPN—Licensed Practical Nurse, EHR—Electronic Health Record, TTS—Tobacco Treatment Specialist, TTP—Tobacco Treatment Program, Med—Medium.

**Table 2:** UVA C3I Tobacco Treatment Program Tier System.

Tier	Criteria	Tobacco Treatment Services Received
1	Impending surgery, or treatment requires cessation (survivor is not eligible for surgery if not tobacco free), mental health diagnoses, or substance abuse history	1 h initial visit with TTS 8-10 phone or in-person visits (~30 min each) Pharmacotherapy (collaboration with UVA pharmacy) Provided information about community resources TTS meets with survivor post-surgery for continued treatment (if applicable)
2	Tobacco user who would like to participate in the TTP and is ready to quit now	1 h initial visit with TTS 4 phone or in-person visits (~30 min each) Pharmacotherapy (collaboration with UVA pharmacy) Provided information about community resources
3	Tobacco user who would like to participate in the TTP but is not ready to quit now	30 min initial visit with TTS TTS provides their contact information Provided information about community resources

Note: TTP—Tobacco Treatment Program, TTS—Tobacco Treatment Specialist, UVA—University of Virginia, C3I—Cancer Center Cessation Initiative.
